# Missense variant in *LOXHD1* is associated with canine nonsyndromic hearing loss

**DOI:** 10.1007/s00439-021-02286-z

**Published:** 2021-05-13

**Authors:** Marjo K. Hytönen, Julia E. Niskanen, Meharji Arumilli, Casey A. Brookhart-Knox, Jonas Donner, Hannes Lohi

**Affiliations:** 1 Department of Medical and Clinical Genetics, University of Helsinki, Helsinki, Finland; 2 Folkhälsan Research Center, Helsinki, Finland; 3 Department of Veterinary Biosciences, University of Helsinki, Helsinki, Finland; 4 Genoscoper Laboratories, Helsinki, Finland; 5 Wisdom Health, Vancouver, WA USA

## Abstract

Hearing loss is a common sensory deficit in both humans and dogs. In canines, the genetic basis is largely unknown, as genetic variants have only been identified for a syndromic form of hearing impairment. We observed a congenital or early-onset sensorineural hearing loss in a Rottweiler litter. Assuming an autosomal recessive inheritance, we used a combined approach of homozygosity mapping and genome sequencing to dissect the genetic background of the disorder. We identified a fully segregating missense variant in *LOXHD1,* a gene that is known to be essential for cochlear hair cell function and associated with nonsyndromic hearing loss in humans and mice. The canine *LOXHD1* variant was specific to the Rottweiler breed in our study cohorts of pure-bred dogs. However, it also was present in some mixed-breed dogs, of which the majority showed Rottweiler ancestry. Low allele frequencies in these populations, 2.6% and 0.04%, indicate a rare variant. To summarize, our study describes the first genetic variant for canine nonsyndromic hearing loss, which is clinically and genetically similar to human *LOXHD1*-related hearing disorder, and therefore, provides a new large animal model for hearing loss. Equally important, the affected breed will benefit from a genetic test to eradicate this *LOXHD1*-related hearing disorder from the population.

**Supplementary Information:**

The online version contains supplementary material available at 10.1007/s00439-021-02286-z.

## Introduction

Hearing loss (HL) is the most common sensory impairment and consequently constitutes a major medical issue, affecting 1–3/1000 newborns and becoming more prevalent by age (Morton and Nance 2006). It is a both clinically and genetically heterogeneous disorder: the severity of the hearing impairment varies and the age of onset ranges from congenital and early-onset to late-onset. Other symptoms may accompany hearing impairment, but nonsyndromic hearing loss (NSHL) is the predominant type. Genetic factors contribute to more than half of the congenital and early childhood nonsyndromic forms, and variants have been reported in over 100 genes to date (https://hereditaryhearingloss.org/). Despite the accumulating knowledge, a large number of patients remain without a molecular diagnosis. Thus, there is a need to better understand the molecular underpinnings of hearing loss and the associated genes and variants in cochlear biology and disease pathophysiology.

The molecular genetics of hearing loss can be addressed by studying spontaneous hearing defects in purebred dogs. Over the past century, selective breeding of dogs has led to hundreds of breeds with specific genomic architectures and the formation of genetic isolates, which can significantly facilitate gene discovery in small study cohorts (Lindblad-Toh et al. 2005). Both syndromic and nonsyndromic congenital as well as adult-onset hearing loss have been reported in dogs across breeds (Strain. 2015), yet their genetic background remains mostly uncharacterized. The most common form of hearing loss in dogs is pigment-associated congenital sensorineural deafness, which occurs in breeds with a lack of pigmentation or piebaldism (De Risio et al. 2016; Comito et al. 2012; Platt et al. 2006; Strain 2004). The trait is associated with regulatory variants in melanocyte inducing transcription factor (*MITF*) (Karlsson et al. 2007). Another syndromic type of HL is a recessive congenital deafness and vestibular syndrome observed in Doberman Pinschers, which is linked to variants in myosin VIIA (*MYOA7*) and tyrosine phosphatase, receptor type Q (*PTPRQ*) (Webb et al. 2019; Guevar et al. 2018). In addition to the congenital HL forms, an adult-onset hearing defect has been reported in Border Collies and mapped to canine chromosome 6 (Yokoyama et al. 2012). In our study, we observed sensorineural hearing loss in Rottweilers and utilized genome-wide array genotyping and genome sequencing to identify a fully segregating novel missense variant in *LOXHD1*, a known hearing loss gene in human.

## Materials and methods

### Study cohorts

EDTA blood samples were collected from 585 privately owned Rottweiler dogs, including four affected littermates. The samples were stored at − 20 °C until genomic DNA was extracted using a semi-automated Chemagen extraction robot (PerkinElmer Chemagen Technologie GmbH). DNA concentration was determined either with NanoDrop ND-1000 UV/Vis Spectrophotometer or Qubit 3.0 Fluorometer (Thermo Fisher Scientific Inc.). Sample collection was ethically approved by the Animal Ethics Committee of State Provincial Office of Southern Finland (ESAVI/343/04.10. 07/2016 and ESAVI/25696/2020).

### Homozygosity mapping

Four affected littermates, one unaffected littermate and two unrelated unaffected Rottweilers were genotyped using the CanineHD Whole-Genome Genotyping BeadChip containing 173,662 markers (Lincoln, NE, USA). Pre-analytical QC was conducted using PLINK (version 1.96b6.20, Chang et al. 2015) and included pruning for sample call rate of > 95%, marker call rate of > 95%, and Hardy–Weinberg equilibrium p value < 1 × 10^–8^. One of the cases was discarded due to a poor call rate. As recommended by Meyermans et al. (2020), pruning for minor allele frequency or LD was not performed. After QC, three cases and three controls, as well as 154,235 markers remained for analysis.

Detection of runs of homozygosity (ROH) was also performed with PLINK 1.9 (Chang et al. 2015). Two rounds of analyses were conducted: first, the optimization of population-dependent parameters using simulated data; and second, detection of ROH shared by the affected dogs. In both analyses, the minimum marker size for ROH (--homozyg-snp) was set to 70 based on the formula described by Purfield et al. (2012) with *α* = 0.05, *N*_s_ = 154,235, *N*_i_ = 6 and mean SNP heterozygosity = 0.21 as evaluated using an in-house Python script. Furthermore, the parameters --homozyg-window-snp, --homozyg-window-missing, --homozyg-window-het, --homozyg-window-threshold and --homozyg-kb were set to a fixed value depending on the analysis (Table 1).

**Table 1:** Values used for parameter optimization and detection of shared ROH in PLINK

Parameter	Values by analysis type	
	Parameter optimization	Detection of shared ROH
--homozyg-window-snp	20	50
--homozyg-window-missing	1	1
--homozyg-window-het	0	1
--homozyg-window-threshold	0.05	0.05
--homozyg-snp	70	70
--homozyg-kb	1000 kb	1000 kb
--homozyg-gap	2000 kb (when unvaried) 20–1000 kb (when varied)	200 kb
--homozyg-density	200 kb/snp (when unvaried) 10–125 kb/snp (when varied)	30 kb/snp

Finally, to establish suitable values for ROH density and maximum gap, genotypes for a fully homozygous individual were simulated based on the population map file and analyzed for maximal genome coverage as described by Meyermans et al. (2020). To calculate maximum genome coverage for the simulated genome, --homozyg-gap was set to 2000 kb and --homosyg-density to 200 kb/snp; genome coverage was determined as the total length of the resulting ROH. The simulated genome was then analyzed by varying --homozyg-density from 10 to 125 kb/snp in increments of 5 kb and --homozyg-gap from 20 to 1000 kb in increments of 20 kb (Table 1).

Based on the simulation, density was set to 30 kb/snp and maximum gap to 200 kb, as genome coverage reached 100% for --homozyg-density at 30 and increased only negligibly for --homozyg-gap after 200. Using these parameters, detection of ROH was performed with --homozyg-group. ROH included in further analyses were required to be allelically shared by all three cases and either allelically different or absent in the controls.

### Whole-exome and -genome sequencing

Whole-exome sequencing (WES) was carried out for one affected dog of the initially identified Rottweiler litter. The exome library was prepared with 140702_canFam3_exomeplus_BB_EZ_HX1 kit with a capture size of 152 Mb from the Roche NimbleGen SeqCap EZ target enrichment design (Broeckx et al. 2015). The library was sequenced with the Illumina NextSeq500 platform with a read length of 300 bp (paired-end reads, 2 × 150 bp) and a coverage of 38× at the Biomedicum Functional Genomics Unit (University of Helsinki, Finland). The exome sequence data analysis, including quality control, mapping, alignment post-processing, single nucleotide variant calling and small indel calling, was performed as described previously (Hytönen et al. 2019). Whole-genome sequencing (WGS) was conducted on another affected Rottweiler from the same litter on Illumina HiSeq2000 high-throughput sequencing platform with a read length of 100 bp (paired-end reads, 2 × 100 bp) and a coverage of 15× at the University of Bern. Reads from the WGS sample were processed using SpeedSeq open-source software with bwa (v0.7.15) for alignment, SAMBLASTER for marking duplicate reads, sorting and BAM compression using Sambamba (Chiang et al. 2015).

For WGS sample, the variant calling of single nucleotide variants (SNVs) and small insertions and deletions (indels) was done using the HaplotypeCaller in gVCF mode (v3.7-0), which is combined with gVCFs from our cohort with combineGVCFs and joint genotyping was done by GenotypeGVCFs in Genome Analysis Tool Kit GATK version 4.1 (McKenna et al. 2010). Mobile Element Locator Tool (MELT) was used to detect mobile element insertions (Gardner et al. 2017) and the reference sequences of the transposons for mobile element insertion (MEI) discovery were retrieved from the Repbase database (Jurka et al. 2005). The DELLY software was used to detect structural variants (SVs), including deletions, duplications, inversions and insertions by independent commands (Rausch et al. 2012). Functional annotation of variants from both WES and WGS samples was done using Ensembl release100 and NCBI *Canis lupus familiaris* Annotation Release 105.

The aligned bam files were submitted to SRA with the BioProject accession PRJNA702911. The sample accession for the exome sample is SAMN17983069 and SAMN17983068 for the WGS sample.

NCBI transcript XM_022421426.1 and UniProt sequence J9PAE4 were used to count the nucleotide and amino acid positions for LOXHD1.

### Variant analysis

The identified variants were imported into a webGQT variant server deployed locally on in-house canine variant datasets for inheritance model-based candidate variant filtering (Arumilli et al. 2020). The variant data of the affected dogs were filtered against 637 control genomes (Online Resource 1) when filtering SNVs and indels, assuming an autosomal recessive inheritance of the disease and allowing a maximum of two heterozygous calls for each variant in the controls. The WGS data of one affected dog were filtered against 290 control genomes (Online Resource 1) when filtering for MEIs and SVs. For SNVs and indels, both cases were required to share the variants in the homozygous state; for SVs and MEIs, only the WGS case was used and both homozygous and heterozygous calls were included to allow for inaccuracies in calling. Prediction of the variant pathogenicity was assessed using PROVEAN (Choi and Chan 2015; Choi et al. 2012) and Poly-Phen2 software (Adzhubei et al. 2010). Finally, homology analysis of a candidate causal variant in the *LOXHD1* gene was performed by retrieving orthologous genes for XP_022277134.1 with NCBI’s blastp web interface and aligning them with COBALT (Johnson et al. 2008; Papadopoulos and Agarwala 2007).

### Genomic DNA analysis

Genotyping of individual dogs was performed with PCR followed by Sanger sequencing. The DNA template was amplified using a forward primer (5′–GCTGTGTGTTGGAGAAGCAA–3′) and a reverse primer (5′–TAGTTGCCTGACACCCTGAG–3′) flanking the *LOXHD1* variant with Taq polymerase (Biotools B&M Labs, S.A.). The products were directly sequenced using the PCR primers on an ABI 3730 capillary sequencer (Life Technologies) after treatment with exonuclease I (New England Biolabs) and rapid alkaline phosphatase (Roche Diagnostics). The Sanger sequence data were analyzed using either Sequencher 5.4 (GeneCodes) or Unipro UGENE v1.32.0 (Rose et al. 2019; Okonechnikov et al. 2012; Golosova et al. 2014).

A sample of 28,116 dogs, including 374 different breeds (Online Resource 7), was submitted for commercial genetic testing at Genoscoper Laboratories (Wisdom Health Finland) during 2017–2020. Another study sample of 771,864 mixed-breed dogs was screened using Wisdom Panel™ (Wisdom Health, WA, USA) genetic testing, including breed detection assessment, during 2019–2021.

## Results

Sensorineural bilateral deafness was diagnosed in four Rottweiler siblings (one female and three males) in a litter of ten puppies using brainstem auditory evoked response (BAER) testing. BAER testing was performed either at 4 (*n* = 2), 5, or 19 months of age, and no auditory response was detected in any of them. However, owners’ observations suggested that the puppies had already been affected by hearing impairment at a few weeks of age. No other clinical signs were observed.

**Fig. 1:**
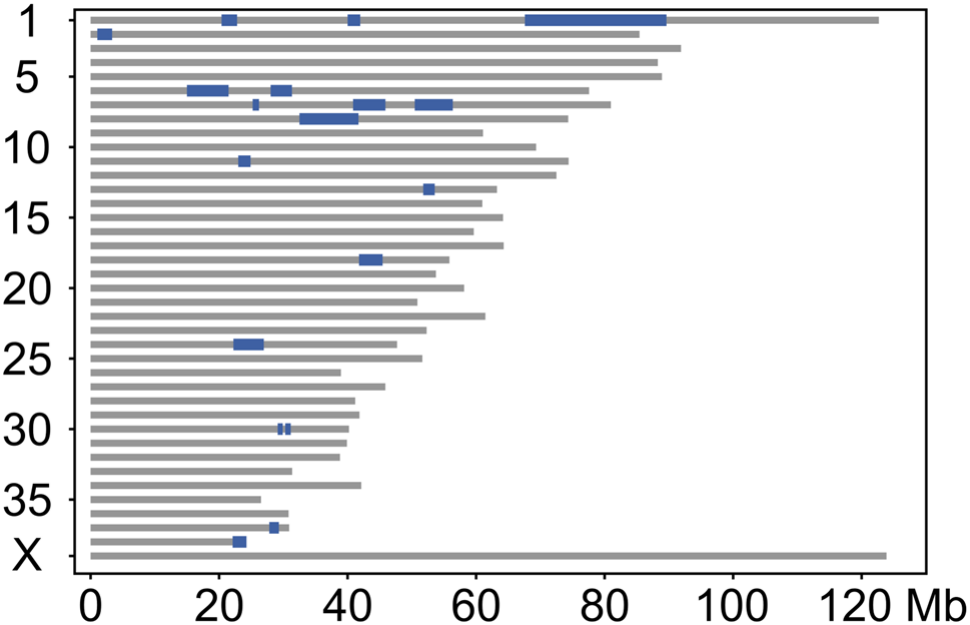
Results of homozygosity mapping in three affected and three unaffected dogs. Case-specific, allelically matching ROH are indicated in blue

We carried out a genome-wide analysis to identify candidate loci using three affected and three unaffected dogs. Homozygosity mapping resulted in 22 regions of case-specific, allelically matching runs of homozygosity (Fig. 1 and Online Resource 2). The regions spanned a total length of 62.3 Mb, the largest being 21.3 Mb on chromosome 1 at 67,943,666–89,271,008.

Subsequently, we performed whole exome sequencing on one affected dog and, later, whole genome sequencing on another case. As a result of variant filtering, 32 homozygous SNVs and indels shared by the two affected dogs were discovered; of these, six were located in case-specific ROH (Table 2, Online Resource 3). Second, 63 SVs and 32 MEIs called either homozygous or heterozygous and private to the sequenced affected dog were identified, and only one MEI resided in case-specific ROH (Table 2, Online Resource 4–5).

**Table 2:** Case-specific SNVs and indels of two affected dogs (one WGS and one WES) and SVs and MEIs of one affected dog (WGS)

Variants	SNVs and indels (N)		SVs (N)		MEIs (N)	
	All	In ROH	All	In ROH	All	In ROH
Total	32	6	63	0	32	1
Exonic	10	2	5	0	0	0
Intronic	13	3	18	0	6	0
Splicing	0	0	0	0	1	0
UTR	4	1	1	0	0	0
Other	5	0	39	0	25	1

The variants were categorized according to NCBI Annotation Release 105

*SNV* single nucleotide variant, *indel* small insertion or deletion, *SV* structural variant, *MEI* mobile element insertion, *ROH* runs of homozygosity

**Fig. 2:**
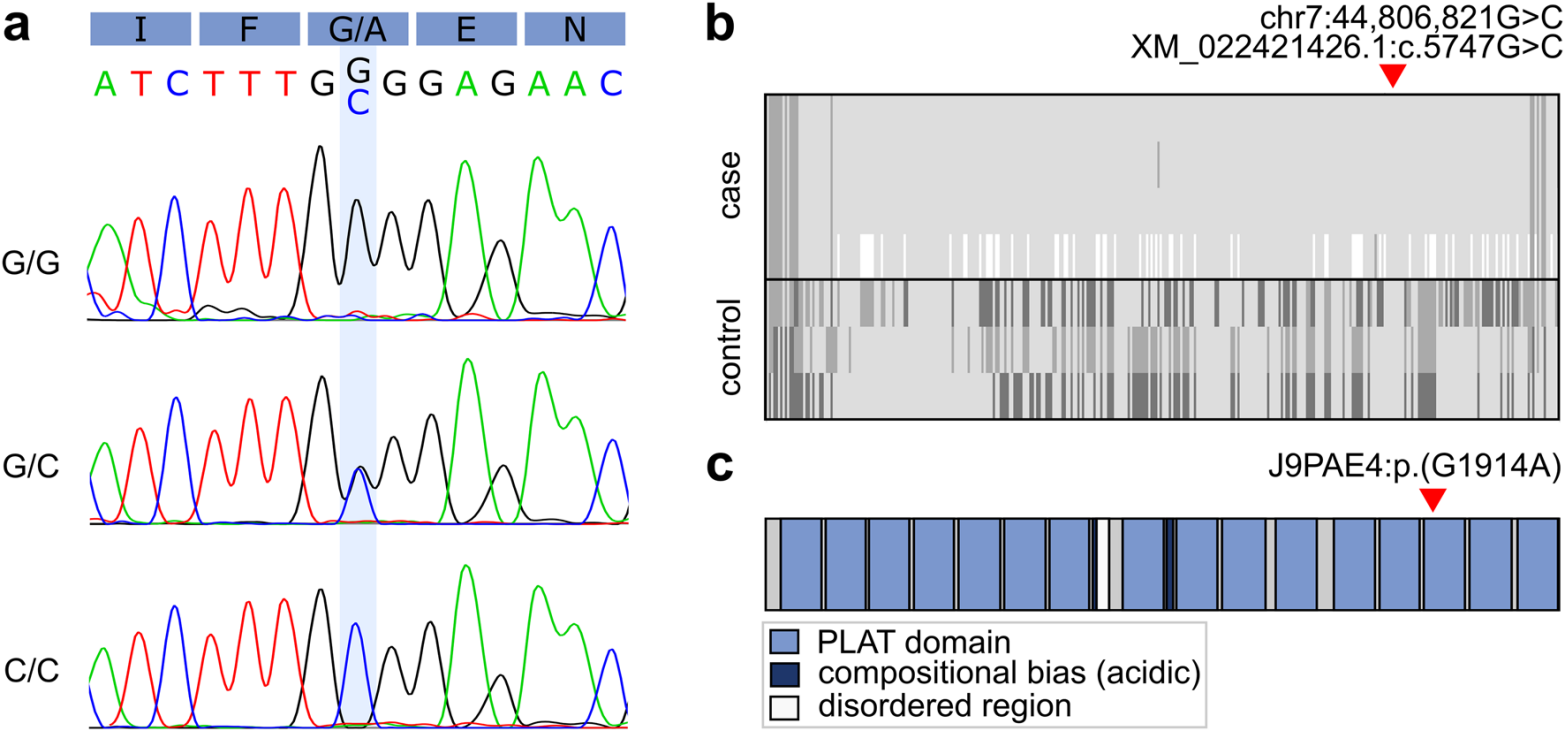
**a** Example chromatograms from Sanger sequencing of the chr7:44,806,821G>C variant. **b** Genotypes of four cases and three controls at a ROH at chr7:41.0–45.7 Mb. A distinct case-specific homozygous haplotype can be observed at 41.2–45.5 Mb. The bottommost case was not included in ROH detection due to a poor call rate. **c** Schematic illustration of the domain structure of LOXHD1 (J9PAE4)

Of the seven case-specific variants that resided in ROH, two exonic variants were considered for further analyses. First, a G>C missense variant at chr7:44,806,821 in lipoxygenase homology domains 1 (*LOXHD1*), a gene that is known to cause hearing loss in humans and mice (Grillet et al. 2009), was predicted to result in a glycine-to-alanine substitution (Fig. 2). Similarly, a C>T missense variant at chr24:25,785,932 in maestro heat like repeat family member 8 (*MROH8*) was predicted to cause a glycine-to-serine substitution. We assessed the pathogenicity of these variants in silico using two protein prediction tools, PROVEAN and PolyPhen-2. First, PROVEAN predicted both the *LOXHD1* and *MROH8* substitutions as “deleterious” with a score of − 4.517 and − 3.336, respectively. Similarly, PolyPhen-2 predicted the *LOXHD1* variant as “probably damaging” with a HumVar score of 0.992 and the *MROH8* variant as “possibly damaging” with a score of 0.550. As *MROH8* has been associated with red blood cell volume, body mass index, telomere length and hippocampal atrophy in humans (Buniello et al. 2019) and its predicted impact was less pathogenic, making it an unlikely candidate variant, we focused on *LOXHD1*. The chr7:44,806,821G>C variant is predicted to cause a glycine-to-alanine substitution p.(G1914A) in the fourteenth PLAT domain of the canine LOXHD1 protein (Fig. 2**c**).

In addition, we assessed the conservation of the G1914 residue with multiple alignment of 99 Eutherian LOXHD1 protein orthologs, including the dog (Online Resource 6). In these species, the glycine residue and several flanking amino acids are fully conserved.

To validate the *LOXHD1* variant, we genotyped it in a cohort of 585 Rottweilers, including the four affected siblings and 581 unaffected dogs. We observed complete segregation of the variant with hearing loss, as all four affected dogs were homozygous for the variant. The unaffected dogs were either heterozygous (*n* = 33) or wild-type (*n* = 548). The six unaffected littermates of the probands were either wild-type or heterozygous for the variant. The allele frequency in the population, excluding the affected family, was 2.6% and carrier frequency 5.3%.

An additional sample of dogs submitted for commercial genetic testing was screened for the *LOXHD1* variant to explore its distribution across breeds. All 28,116 tested dogs representing 374 breeds, breed varieties or designer dog mixes were found homozygous for the wild-type allele (Online Resource 7). Finally, the variant was also screened in a larger study sample of 771,864 dogs submitted to genetic testing, including breed detection assessment. A variant carrier frequency of 0.08% and allele frequency of 0.04% were observed in this dataset. Interestingly, six dogs were found homozygous for the *LOXHD1* variant. We were able to contact the owners of 4/6 of the homozygous dogs and the owners reported profound hearing loss or deafness in all of them. One of the deaf dogs did not show any immediate Rottweiler ancestry, while one was a purebred Rottweiler and two were mixed-breed with Rottweiler ancestry. Altogether, of the dogs carrying at least one copy of the deafness candidate variant, 63.4% showed evidence of Rottweiler ancestry in their immediate three-generation pedigree going back to great-grandparents, providing further support for a link between this specific breed background and the presence of the variant.

According to a study by Riazuddin et al. (2012), heterozygous *LOXHD1* variants in humans have been suggested to contribute to autosomal dominant late-onset Fuchs endothelial corneal dystrophy (FECD). For this reason, we assessed the eye examinations performed as a part of the breeding programs and regular health check-ups of the Rottweilers genotyped either homozygous or heterozygous for the *LOXHD1* variant. Two of the homozygous deaf dogs had been eye examined healthy, one at 2 years and the other at 8 years old. In addition, eye examination reports were available for 22/33 heterozygous dogs, examined between 1 and 7 years and 4 months old. Three heterozygous dogs were diagnosed with different forms of cataract, which is a relatively common eye disease in Rottweilers. No signs of corneal dystrophy were reported in any of the dogs.

## Discussion

We describe here a missense variant in *LOXHD1* associated with an autosomal recessive congenital nonsyndromic hearing loss in Rottweilers. The variant is rare, yet we confirmed it to be fully segregating with the disease in the breed. This is the first genetic defect identified for NSHL in dogs.

The dogs with the identified *LOXHD1* variant had either congenital or early-onset hearing loss. The owners’ reports suggest that the puppies had at least some hearing impairment already at a few weeks of age. However, the confirmed diagnosis by BAER was acquired later and by that time, the hearing loss was total. Therefore, it is likely that the hearing impairment was congenital and progressed to deafness in a few months, although this remains unconfirmed. The hearing loss in Rottweilers can be defined as nonsyndromic as the affected dogs showed no other consistent clinical features.

Congenital deafness has been reported in Rottweilers previously as sporadic cases (Coppens et al. 2001; Strain 1996). Histopathological examination of one 4.5-month-old bilaterally deaf Rottweiler puppy demonstrated severe degeneration of hair cells and spiral ganglion while the vestibular organ was unaffected (Coppens et al. 2001). Strikingly similar changes are seen in the *samba* mouse line generated in an ethylnitrosourea (ENU) mutagenesis screen, where a *Loxhd1* missense variant leads to hearing loss (Grillet et al. 2009). Stereociliary development in *samba* mice is normal, but hair cell function is altered by postnatal day 21 and hair cells eventually undergo degeneration followed by possibly secondary loss of spiral ganglion neurons. Based on the similarities of the canine and murine models, it is probable that the histopathological changes described in the previous Rottweiler cases represent the canine *LOXHD1* c.1914G>A variant identified in this study. It can be concluded that *LOXHD1* has an essential role in maintaining normal cochlear hair cell function. Interestingly, murine *Loxhd1* is mainly expressed in the membrane of mature mechanosensory hair cells in the cochlea and high levels in testis, an organ also enriched with stereocilia. Its numerous PLAT (polycystin/lipoxygenase/alpha-toxin) domains are likely involved in targeting the protein to the plasma membrane (Grillet et al. 2009; Bateman and Sandford 1999), but the specific function of LOXHD1 in ciliary structures remains unclear.

In humans, several variants in *LOXHD1* have been reported to lead to autosomal recessive nonsyndromic hearing loss (ARNSHL) (DFNB77; OMIM #613079) (Bai et al. 2020; Maekawa et al. 2019; Shen et al. 2019; Zhang et al. 2019; Mori et al. 2015; Riazuddin et al. 2012; Edvardson et al. 2011; Grillet et al. 2009). The severity of the patients’ hearing impairment depends on the genetic defect and varies from mild to profound and is either stable or progressive. Moreover, the age of onset ranges from congenital to even adulthood. Interestingly, one of the human variants, p.(G1849R), is homologous in position with the canine p.(G1914A) substitution. The patient, who was compound heterozygous for *LOXHD1* with the p.(G1849R) and a p.(Y1541*) variant, had been diagnosed with severe congenital hearing loss at the age of two years (Plevova et al. 2017). The homology of the human and canine variant further suggests that substitution of the glycine residue interferes with the function of LOXHD1.

In addition to nonsyndromic hearing loss, an enrichment of *LOXHD1* variants in patients affected by Fuchs endothelial corneal dystrophy (FECD) has been observed in one study, suggesting that *LOXHD1* could be relevant to the FECD pathogenesis (Riazuddin et al. 2012). Conversely, other studies have detected no association between *LOXHD1* variants and FECD in the HL patients and their relatives (Bai et al. 2020; Wesdorp et al. 2018). Therefore, the link between FECD and *LOXHD1* is yet unconfirmed, with a need for further evidence. We did not observe signs of corneal dystrophy in any of the eye examined dogs either homozygous or heterozygous for the *LOXHD1* variant. However, we cannot exclude the possibility of later onset clinical signs.

In conclusion, we describe a rare novel missense variant in *LOXHD1* associated with canine autosomal recessive nonsyndromic hearing loss, providing a new animal model for human hearing disorders. The variant was observed to be specific to the Rottweiler breed. The affected breed will benefit from a genetic test to eradicate the hearing impairment from the population.

## Supplementary Information

Below is the link to the electronic supplementary material.

Supplementary file1 (XLSX 11 kb)

Supplementary file2 (XLSX 10 kb)

Supplementary file3 (XLSX 15 kb)

Supplementary file4 (XLSX 18 kb)

Supplementary file5 (PDF 132 kb)

Supplementary file6 (XLSX 14 kb)

Supplementary file7 (XLSX 19 kb)

## Data Availability

Exome and whole genome sequencing data from three dogs have been published in the SRA under the BioProject accession number PRJNA702911 https://www.ncbi.nlm.nih.gov/sra/?term=PRJNA702911.
