# Supplementary material for: Missense variant in LOXHD1 is associated with canine nonsyndromic hearing loss
Source: Hum Genet. 2021 May 13;140(11):1611–8. doi: 10.1007/s00439-021-02286-z (PMC8521602; doi:10.1007/s00439-021-02286-z)
Supplement: Supplementary file 5 — Supplementary file5 (PDF 132 kb) [file 439_2021_2286_MOESM5_ESM.pdf]

Online Resource 6. Amino acid sequences of 99 Eutherian *LOXHD1* orthologs flanking the canine p.(G1914A) variant site (indicated in blue).

Sequence ID	Scientific name	Common name	Amino acid sequence
XP_022277134.1	*Canis lupus familiaris*	Domestic dog	TDANVFIIIFGENGDSGTLAL
XP_025284772.2	*Canis lupus dingo*	Dingo	TDANVFIIIFGENGDSGTLAL
XP_035920209.1	*Lynx canadensis*	Canada lynx	TDANVFIIIFGENGDSGTLAL
XP_039084791.1	*Hyaena hyaena*	Striped hyena	TDANVFIVIFGENGDSGTLAL
XP_035579822.1	*Zalophus californianus*	California sealion	TDANVYIIIFGENGDSGTLAL
XP_032278619.1	*Phoca vitulina*	Harbor seal	TDANVFIIIFGENGDSGTLAL
XP_025852839.1	*Vulpes vulpes*	Red fox	TDANVFIIIFGENGDSGTLAL
XP_035294917.1	*Cricetulus griseus*	Chinese hamster	TDANVFIIIFGENGDSGTLAL
XP_037052841.1	*Peromyscus leucopus*	White-footed mouse	TDANVFIIIFGENGDSGTLAL
XP_032326338.1	*Camelus ferus*	Wild bactrian camel	TDANVFIIIFGENGDSGTLAL
XP_036679115.1	*Balaenoptera musculus*	Blue whale	TDANVFIIIFGENGDSGTLAL
XP_019319691.1	*Panthera pardus*	Leopard	TDANVFIIIFGENGDSGTLAL
XP_036085909.1	*Rousettus aegyptiacus*	Egyptian rousette	TDANVFIIIFGENGDSGTLAL
XP_038187071.1	*Arvicola amphibius*	Eurasian water mole	TDANVFIIIFGENGDSGTLAL
XP_023386674.1	*Pteropus vampyrus*	Large flying fox	TDANVFIIIFGENGDSGTLAL
XP_037364471.1	*Talpa occidentalis*	Iberian mole	TDANVFIIIFGENGDSGTLAL
XP_032166523.1	*Mustela erminea*	Ermine	TDANVFIIIFGENGDSGTLAL
XP_036061122.1	*Onychomys torridus*	Southern grasshopper mouse	TDANVFIIIFGENGDSGTLAL
XP_039084792.1	*Hyaena hyaena*	Striped hyena	TDANVFIVIFGENGDSGTLAL
XP_006526497.1	*Mus musculus*	Mouse	TDANVFIIIFGENGDSGTLAL
VFV36830.1	*Lynx pardinus*	Iberian lynx	TDANVFIIIFGENGDSGTLAL
XP_022351677.1	*Enhydra lutris kenyoni*	Sea otter	TDANVFIIIFGENGDSGTLAL
XP_011930055.1	*Cercocebus atys*	Sooty mangabey	TDANVFIIIFGENGDSGTLAL
XP_032715367.1	*Lontra canadensis*	River otter	TDANVFVIIFGENGDSGTLAL
XP_031297524.1	*Camelus dromedarius*	Dromedary	TDANVFIIIFGENGDSGTLAL
XP_014977356.2	*Macaca mulatta*	Rhesus macaque	TDANVFIIIFGENGDSGTLAL
NP_001371403.1	*Homo sapiens*	Human	TDANVFIIIFGENGDSGTLAL
XP_011770643.1	*Macaca nemestrina*	Pig-tailed macaque	TDANVFIIIFGENGDSGTLAL
XP_026352032.1	*Ursus arctos horribilis*	Grizzly bear	TDANVFIIIFGENGDSGTLAL
XP_015295465.1	*Macaca fascicularis*	Crab-eating macaque	TDANVFIIIFGENGDSGTLAL
KFO34372.1	*Fukomys damarensis*	Damaraland mole rat	TDANVFIIIFGENGDSGTLAL
XP_017807186.2	*Papio anubis*	Olive baboon	TDANVFIIIFGENGDSGTLAL
XP_017651795.1	*Nannospalax galili*	Northern Israeli blind mole rat	TDANVFIIIFGENGDSGTLAL
XP_021542012.1	*Neomonachus schauinslandi*	Hawaiian monk seal	TDANVFIIIFGENGDSGTLAL
XP_027954221.1	*Eumetopias jubatus*	Steller sea lion	TDANVYIIIFGENGDSGTLAL
XP_008259480.1	*Oryctolagus cuniculus*	Rabbit	TDANVFIIIFGENGDSGTLPL
XP_016789110.2	*Pan troglodytes*	Chimpanzee	TDANVFIIIFGENGDSGTLAL
KAB0377950.1	*Muntiacus reevesi*	Reeves' muntjac	TDANVFIIIFGENGDSGTLAL
XP_007972328.2	*Chlorocebus sabaeus*	Green monkey	TDANVFIIIFGENGDSGTLAL
XP_034799501.1	*Pan paniscus*	Pygmy chimpanzee	SDANVFIIIFGENGDSGTLAL
XP_027432285.2	*Zalophus californianus*	California sealion	TDANVYIIIFGENGDSGTLAL
XP_004408596.1	*Odobenus rosmarus divergens*	Pacific walrus	TDANVYIIIFGENGDSGTLAL
XP_035126836.1	*Callithrix jacchus*	White-tufted-ear marmoset	TDADVFIIIFGENGDSGTLAL
XP_008689836.1	*Ursus maritimus*	Polar bear	TDANVFIIIFGENGDSGTLAL
XP_037594912.1	*Cebus imitator*	Panamanian white-faced capuchin	TDANVFIIIFGENGDSGTLAL
KAB0361263.1	*Muntiacus muntjak*	Barking deer	TDANVFIIIFGENGDSGTLAL
XP_020948244.1	*Sus scrofa*	Pig	TDANVFIIIFGENGDSGTLAL
KAF4024825.1	*Cervus hanglu yarkandensis*	Yarkand deer	TDANVFIIIFGENGDSGTLAL
XP_023096968.1	*Felis catus*	Cat	TDANVFIIIFGENGDSGTLAL
XP_007080621.1	*Panthera tigris altaica*	Siberian tiger	TDANVFIIIFGENGDSGTLAL

KAF0874837.1	*Crocuta crocuta*	Spotted hyena	TDANVFIIIFGENGDSGTLAL
XP_036874966.1	*Manis javanica*	Malayan pangolin	TDANVFITIFGENGDSGTLAL
XP_007639941.1	*Cricetulus griseus*	Chinese hamster	TDANVFIIIFGENGDSGTLAL
XP_004752399.1	*Mustela putorius furo*	European domestic ferret	TDANVFIIIFGENGDSGTLAL
XP_016789112.2	*Pan troglodytes*	Chimpanzee	TDANVFIIIFGENGDSGTLAL
XP_037662141.1	*Choloepus didactylus*	Southern two-toed sloth	TDANVFIIIFGENGDSGTLPL
XP_026924181.1	*Acinonyx jubatus*	Cheetah	TDANVFIIIFGENGDSGTLAL
XP_016789111.2	*Pan troglodytes*	Chimpanzee	TDANVFIIIFGENGDSGTLAL
XP_025742588.1	*Callorhinus ursinus*	Northern fur seal	TDANVYIIIFGENGDSGTLAL
XP_014684407.1	*Equus asinus*	Donkey	TDANVFIIIFGENGDSGTLAL
XP_024436389.1	*Desmodus rotundus*	Vampire bat	TDANVFIIIFGENGDSGTLAL
XP_034851601.1	*Mirounga leonina*	Southern elephant seal	TDANVFIIIFGENGDSGTLAL
XP_016080606.1	*Miniopterus natalensis*	Natal long-fingered bat	TDANVFITIFGENGDSGTLAL
XP_004422559.1	*Ceratotherium simum simum*	Southern white rhinoceros	TDANVFIIIFGENGDSGILPL
XP_027253746.1	*Cricetulus griseus*	Chinese hamster	TDANVFIIIFGENGDSGTLAL
XP_006144481.1	*Tupaia chinensis*	Chinese tree shrew	TDANVFIIIFGENGDSGTLAL
XP_006973912.1	*Peromyscus maniculatus bairdii*	Prairie deer mouse	TDANVFIIIFGENGDSGTLAL
XP_028727861.1	*Peromyscus leucopus*	White-footed mouse	TDANVFIIIFGENGDSGTLAL
XP_002918168.1	*Ailuropoda melanoleuca*	Giant panda	TDANVFIIIFGENGDSGTLAL
XP_032741568.1	*Rattus rattus*	Black rat	TDANVFIIIFGENGDSGTLAL
XP_027816689.1	*Ovis aries*	Sheep	TDANVFIIIFGENGDSGTLAL
NP_001099602.3	*Rattus norvegicus*	Rat	TDANVFIIIFGENGDSGTLAL
XP_024840272.1	*Bos taurus*	Bovine	TDANVFIIIFGENGDSGTLAL
XP_036679116.1	*Balaenoptera musculus*	Blue whale	TDANVFIIIFGENGDSGTLAL
KAF6422944.1	*Rousettus aegyptiacus*	Egyptian rousette	TDANVFIIIFGENGDSGTLAL
XP_017895308.1	*Capra hircus*	Goat	TDANVFIIIFGENGDSGTLAL
XP_038187072.1	*Arvicola amphibius*	Eurasian water mole	TDANVFIIIFGENGDSGTLAL
XP_036061123.1	*Onychomys torridus*	Southern grasshopper mouse	TDANVFIIIFGENGDSGTLAL
XP_011359407.1	*Pteropus vampyrus*	Large flying fox	TDANVFIIIFGENGDSGTLAL
XP_026270296.1	*Urocitellus parryii*	Arctic ground squirrel	TDANVFIIIFGENGDSGTLAL
XP_010840315.1	*Bison bison bison*	Plains bison	TDANVFIIIFGENGDSGTLAL
XP_003474113.1	*Cavia porcellus*	Guinea pig	TDANVFIIIFGENGDSGTLAL
XP_011833467.1	*Mandrillus leucophaeus*	Drill	TDANVFIIIFGENGDSGTLAL
XP_005356346.1	*Microtus ochrogaster*	Prairie vole	TDANVFIIIFGENGDSGTLAL
XP_004579578.1	*Ochotona princeps*	Southern American pika	TDANVFIIIFGENGDSGTLAL
XP_005865803.2	*Myotis brandtii*	Brandt's bat	TDANVFITIFGENGDSGTLAL
NP_653213.6	*Homo sapiens*	Human	TDANVFIIIFGENGDSGTLAL
XP_007465678.1	*Lipotes vexillifer*	Yangtze river dolphin	TDANVFIIIFGENGDSGTLAL
XP_025220970.1	*Theropithecus gelada*	Gelada baboon	TDANVFIIIFGENGDSGTLAL
XP_023063241.1	*Piliocolobus tephrosceles*	Ugandan red colobus	TDANVFIIIFGENGDSGTLAL
XP_028639050.1	*Grammomys surdaster*	African woodland thicket rat	TDANVFIIIFGENGDSGTLAL
XP_029083443.1	*Monodon monoceros*	Narwhal	TDANVFIIIFGENGDSGTLAL
XP_004623679.1	*Octodon degus*	Degu	TDANVFIIIFGENGDSGTLAL
XP_020948242.1	*Sus scrofa*	Pig	TDANVFIIIFGENGDSGTLAL
XP_022417502.1	*Delphinapterus leucas*	Beluga whale	TDANVFIIIFGENGDSGTLAL
XP_004059420.3	*Gorilla gorilla gorilla*	Western lowland gorilla	TDANVFIIIFGENGDSGTLAL
XP_027991752.1	*Eptesicus fuscus*	Big brown bat	TDANVFITIFGENGDSGTLAL
XP_032101129.1	*Sapajus apella*	Brown-capped capuchin	TDANVFIIIFGENGDSGTLAL
XP_005372931.1	*Chinchilla lanigera*	Long-tailed chinchilla	TDANVFIIIFGENGDSGTLAL
			** * ********* * *
